# (1α,2β,3α,7α,11α,13β)-1,3,11-Triacet­oxy-2,13-bis­(benz­yloxy)-7-hydr­oxy-21-methyl-*N*,19-secohetisan-19-al

**DOI:** 10.1107/S1600536808019223

**Published:** 2008-07-05

**Authors:** Shu-Hua Li, Tie-Ying Zi, Xiong-Qing Wang

**Affiliations:** a Department of Chemistry and Life Sciences, Leshan Teachers College, Leshan 614004, People’s Republic of China; b The First Affiliated Hospital, Chengdu Medical College, Xindu 610500, People’s Republic of China; c Mianyang Normal University, Mianyang 621000, People’s Republic of China

## Abstract

The title compound (delgradine), C_41_H_43_NO_12_, is a hetisine-type C_20_-diterpenoid alkaloid, isolated from the roots of *Aconitum carmichaeli* Debx. In the crystal structure, the mol­ecule assumes an U-shaped conformation, the terminal benzene rings being approximately parallel and partially overlapped with each other. The mol­ecule contains eight alicyclic and heterocyclic rings. Cyclo­hexane rings *A* and *B* adopt similar chair conformations; the six-membered rings *C*, *D* and *E* form a bicyclo­[2.2.2]octane system with a boat conformation for each six-membered ring, the six-membered heterocyclic ring *F* has a screw-boat conformation and both of the five-membered rings G and H have envelope conformations. The crystal structure contains inter­molecular O—H⋯O hydrogen bonding.

## Related literature

For related literature, see: Deng *et al.* (1992 ▶).
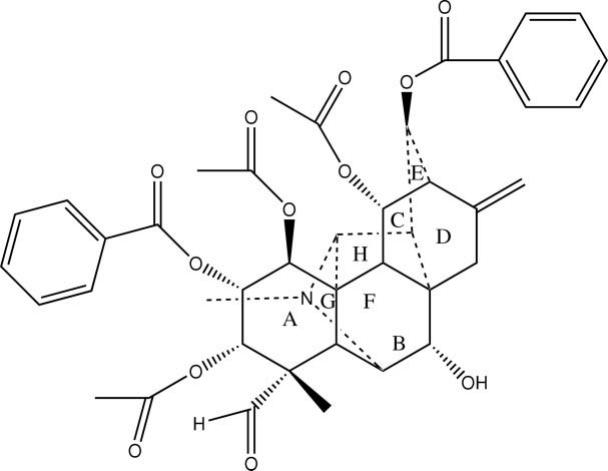


## Experimental

### 

#### Crystal data

C_41_H_43_NO_12_
*M* *_r_* = 741.76
Monoclinic, 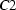

*a* = 19.892 (4) Å
*b* = 11.307 (5) Å
*c* = 16.825 (5) Å
β = 91.07 (2)°
*V* = 3784 (2) Å^3^
*Z* = 4
Mo *K*α radiation
μ = 0.10 mm^−1^
*T* = 291 (2) K
0.42 × 0.40 × 0.36 mm

#### Data collection

Enraf–Nonius CAD-4 diffractometer
Absorption correction: none
4309 measured reflections
3705 independent reflections
2282 reflections with *I* > 2σ(*I*)
*R*_int_ = 0.018
3 standard reflections every 300 reflections intensity decay: 2.6%

#### Refinement

*R*[*F* ^2^ > 2σ(*F* ^2^)] = 0.048
*wR*(*F* ^2^) = 0.130
*S* = 1.01
3705 reflections
498 parameters
1 restraint
H-atom parameters constrained
Δρ_max_ = 0.19 e Å^−3^
Δρ_min_ = −0.20 e Å^−3^

### 

Data collection: *DIFRAC* (Gabe & White, 1993 ▶); cell refinement: *DIFRAC*; data reduction: *NRCVAX* (Gabe *et al.*, 1989 ▶); program(s) used to solve structure: *SHELXS97* (Sheldrick, 2008 ▶); program(s) used to refine structure: *SHELXL97* (Sheldrick, 2008 ▶); molecular graphics: *ORTEP-3 for Windows* (Farrugia, 1997 ▶); software used to prepare material for publication: *WinGX* (Farrugia, 1999 ▶).

## Supplementary Material

Crystal structure: contains datablocks I, global. DOI: 10.1107/S1600536808019223/xu2424sup1.cif

Structure factors: contains datablocks I. DOI: 10.1107/S1600536808019223/xu2424Isup2.hkl

Additional supplementary materials:  crystallographic information; 3D view; checkCIF report

## Figures and Tables

**Table 1:** Hydrogen-bond geometry (Å, °)

*D*—H⋯*A*	*D*—H	H⋯*A*	*D*⋯*A*	*D*—H⋯*A*
O7—H7*A*⋯O19^i^	0.82	1.94	2.743 (5)	167

Symmetry code: (i) 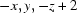.

**Fig. 1.**
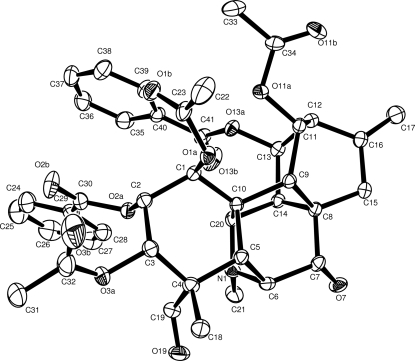
The molecular structure of the title compound with 30% probability displacement ellipsoids for non-H atoms. H atoms have been omitted for clarity.

## supplementary crystallographic information

### Comment

The diterpenoid alkaloid, delgradine, was previously isolated from *Delphinium grandiflorum L*. (Deng *et al.*, 1992), and its structure was established from the spectroscopic data. In our recent investigation, it was isolated from *Aconitum carmichaeli Debx*, and its crystal structure was determined.

The molecular structure of the title compound is shown in Fig. 1. The molecule of the title compound assumes an U-shaped conformation, with terminal benzene rings being approximately parallel and partially overlapped to each other. The molecule contains eight alicyclic and heterocyclic rings. Cyclohexane rings A (C1/C2/C3/C4/C5/C10) and B (C5/C6/C7/C8/C9/C10) adopt chair conformations; six-membered rings C (C8/C9/C11/C12/C13/C14), D (C8/C9/C11/C12/C15/C16) and E (C8/C12/C13/C14/C15/C16) form a bicycle [2.2.2] octane system with the boat conformation for each six-membered ring C, D and E; the six-membered heterocyclic ring F (C6/C7/C8/C14/C20/N1) adopts a screw-boat conformation; while the five-membered rings G (C5/C6/C10/C20/N1) and H (C8/C9/C10/C14/C20) adopt the same envelope conformation.

The crystal structure contains intermolecular O—H···O hydrogen bond between the hydroxy group and aldehyde O atom (Table 1).

### Experimental

The title compound was isolated from the roots of *Aconitum carmichaeli Debx* and crystals suitable for X-ray structure analysis were obtained by slow evaporation from an acetone solution at room temperature.

### Refinement

H atoms were located geometrically with C—H distance of 0.93–0.98 Å, and refined using a riding model with *U*_iso_(H) = 1.2*U*_eq_(C). The absolute configuration has not been determined for the structure.

### Crystal data

**Table:**

C_41_H_43_NO_12_	*F*_000_ = 1568
*M**_r_* = 741.76	*D*_x_ = 1.302 Mg m^−^^3^
Monoclinic, *C*2	Mo *K*α radiation λ = 0.71073 Å
Hall symbol: C 2y	Cell parameters from 55 reflections
*a* = 19.892 (4) Å	θ = 4.7–7.6º
*b* = 11.307 (5) Å	µ = 0.10 mm^−^^1^
*c* = 16.825 (5) Å	*T* = 291 (2) K
β = 91.07 (2)º	Block, colourless
*V* = 3784 (2) Å^3^	0.42 × 0.40 × 0.36 mm
*Z* = 4	

### Data collection

**Table:**

Enraf–Nonius CAD-4 diffractometer	*R*_int_ = 0.018
Radiation source: fine-focus sealed tube	θ_max_ = 25.5º
Monochromator: graphite	θ_min_ = 1.2º
*T* = 291(2) K	*h* = −6→24
ω/2θ scans	*k* = −13→0
Absorption correction: none	*l* = −20→20
4309 measured reflections	3 standard reflections
3705 independent reflections	every 300 reflections
2282 reflections with *I* > 2σ(*I*)	intensity decay: 2.6%

### Refinement

**Table:**

Refinement on *F*^2^	Secondary atom site location: difference Fourier map
Least-squares matrix: full	Hydrogen site location: inferred from neighbouring sites
*R*[*F*^2^ > 2σ(*F*^2^)] = 0.048	H-atom parameters constrained
*wR*(*F*^2^) = 0.130	*w* = 1/[σ^2^(*F*_o_^2^) + (0.0734*P*)^2^] where *P* = (*F*_o_^2^ + 2*F*_c_^2^)/3
*S* = 1.01	(Δ/σ)_max_ = 0.001
3705 reflections	Δρ_max_ = 0.19 e Å^−^^3^
498 parameters	Δρ_min_ = −0.20 e Å^−^^3^
1 restraint	Extinction correction: SHELXL97 (Sheldrick, 2008), Fc^*^=kFc[1+0.001xFc^2^λ^3^/sin(2θ)]^-1/4^
Primary atom site location: structure-invariant direct methods	Extinction coefficient: 0.0010 (3)

### Special details

**Table:**

Geometry. All e.s.d.'s (except the e.s.d. in the dihedral angle between two l.s. planes) are estimated using the full covariance matrix. The cell e.s.d.'s are taken into account individually in the estimation of e.s.d.'s in distances, angles and torsion angles; correlations between e.s.d.'s in cell parameters are only used when they are defined by crystal symmetry. An approximate (isotropic) treatment of cell e.s.d.'s is used for estimating e.s.d.'s involving l.s. planes.
Refinement. Refinement of *F*^2^ against ALL reflections. The weighted *R*-factor *wR* and goodness of fit *S* are based on *F*^2^, conventional *R*-factors *R* are based on *F*, with *F* set to zero for negative *F*^2^. The threshold expression of *F*^2^ > σ(*F*^2^) is used only for calculating *R*-factors(gt) *etc*. and is not relevant to the choice of reflections for refinement. *R*-factors based on *F*^2^ are statistically about twice as large as those based on *F*, and *R*- factors based on ALL data will be even larger.

### Fractional atomic coordinates and isotropic or equivalent isotropic displacement parameters (Å^2^)

**Table:**

	*x*	*y*	*z*	*U*_iso_*/*U*_eq_	
N1	0.10745 (17)	0.5052 (3)	0.8921 (2)	0.0427 (9)	
O1A	0.21024 (15)	0.8335 (3)	0.78232 (16)	0.0456 (8)	
O1B	0.26263 (18)	0.8288 (3)	0.6655 (2)	0.0639 (10)	
O2A	0.12403 (14)	0.5813 (3)	0.69392 (16)	0.0449 (7)	
O2B	0.1133 (2)	0.6310 (4)	0.5646 (2)	0.0872 (13)	
O3A	0.01724 (15)	0.7482 (3)	0.69724 (18)	0.0593 (9)	
O3B	0.0463 (4)	0.9136 (5)	0.6367 (3)	0.128 (2)	
O7	0.14601 (16)	0.5003 (3)	1.06651 (19)	0.0573 (9)	
H7A	0.1100	0.5065	1.0891	0.069*	
O11A	0.33185 (14)	0.7260 (3)	0.82500 (17)	0.0465 (8)	
O11B	0.43602 (17)	0.7575 (4)	0.8731 (2)	0.0794 (12)	
O13A	0.30189 (15)	0.4752 (3)	0.80593 (17)	0.0472 (8)	
O13B	0.2702 (2)	0.2863 (3)	0.7952 (2)	0.0871 (13)	
O19	−0.03068 (16)	0.5601 (4)	0.8561 (2)	0.0716 (11)	
C1	0.1948 (2)	0.7076 (4)	0.7724 (2)	0.0378 (10)	
H1	0.2332	0.6668	0.7492	0.045*	
C2	0.1350 (2)	0.7038 (4)	0.7150 (3)	0.0437 (11)	
H2	0.1462	0.7480	0.6670	0.052*	
C3	0.0741 (2)	0.7595 (4)	0.7516 (2)	0.0463 (11)	
H3	0.0834	0.8440	0.7586	0.056*	
C4	0.0528 (2)	0.7102 (4)	0.8317 (3)	0.0471 (11)	
C5	0.1138 (2)	0.7027 (4)	0.8903 (2)	0.0399 (10)	
H5	0.1200	0.7777	0.9187	0.048*	
C6	0.1016 (2)	0.6019 (5)	0.9487 (3)	0.0482 (12)	
H6	0.0562	0.6059	0.9702	0.058*	
C7	0.1547 (2)	0.5984 (4)	1.0151 (2)	0.0439 (11)	
H7	0.1519	0.6714	1.0462	0.053*	
C8	0.2248 (2)	0.5890 (4)	0.9808 (2)	0.0383 (10)	
C9	0.2367 (2)	0.6898 (4)	0.9200 (2)	0.0374 (10)	
H9	0.2253	0.7655	0.9447	0.045*	
C10	0.1821 (2)	0.6595 (4)	0.8551 (2)	0.0361 (10)	
C11	0.3135 (2)	0.6881 (4)	0.9037 (3)	0.0412 (11)	
H11	0.3342	0.7447	0.9409	0.049*	
C12	0.3439 (2)	0.5665 (4)	0.9241 (3)	0.0462 (12)	
H12	0.3893	0.5599	0.9028	0.055*	
C13	0.2999 (2)	0.4682 (4)	0.8923 (3)	0.0449 (11)	
H13	0.3182	0.3919	0.9099	0.054*	
C14	0.2277 (2)	0.4820 (4)	0.9232 (2)	0.0393 (10)	
H14	0.2133	0.4095	0.9499	0.047*	
C15	0.2779 (2)	0.5792 (5)	1.0474 (2)	0.0485 (12)	
H15A	0.2780	0.6510	1.0790	0.058*	
H15B	0.2670	0.5135	1.0819	0.058*	
C16	0.3465 (2)	0.5605 (4)	1.0133 (3)	0.0489 (12)	
C17	0.4016 (3)	0.5488 (7)	1.0559 (4)	0.086 (2)	
H17A	0.4429	0.5437	1.0311	0.103*	
H17B	0.3995	0.5457	1.1110	0.103*	
C18	−0.0002 (2)	0.7940 (5)	0.8672 (3)	0.0626 (14)	
H18A	−0.0377	0.8012	0.8307	0.075*	
H18B	0.0195	0.8704	0.8761	0.075*	
H18C	−0.0153	0.7623	0.9167	0.075*	
C19	0.0189 (2)	0.5891 (5)	0.8198 (3)	0.0542 (12)	
H19	0.0370	0.5363	0.7836	0.065*	
C20	0.1745 (2)	0.5210 (4)	0.8599 (3)	0.0402 (10)	
H20	0.1800	0.4818	0.8085	0.048*	
C21	0.0857 (3)	0.3858 (5)	0.9132 (3)	0.0620 (14)	
H21A	0.0379	0.3856	0.9208	0.074*	
H21B	0.1082	0.3616	0.9615	0.074*	
H21C	0.0966	0.3321	0.8712	0.074*	
C22	0.2639 (4)	1.0078 (5)	0.7406 (4)	0.092 (2)	
H22A	0.2896	1.0390	0.6977	0.110*	
H22B	0.2899	1.0123	0.7891	0.110*	
H22C	0.2234	1.0531	0.7459	0.110*	
C23	0.2465 (3)	0.8829 (5)	0.7239 (3)	0.0537 (13)	
C24	0.0951 (3)	0.3908 (6)	0.5239 (4)	0.0798 (18)	
H24	0.0961	0.4448	0.4823	0.096*	
C25	0.0857 (4)	0.2712 (8)	0.5086 (5)	0.105 (2)	
H25	0.0815	0.2450	0.4564	0.126*	
C26	0.0826 (3)	0.1918 (7)	0.5692 (5)	0.097 (2)	
H26	0.0760	0.1120	0.5580	0.116*	
C27	0.0890 (3)	0.2280 (6)	0.6458 (4)	0.0789 (18)	
H27	0.0867	0.1735	0.6870	0.095*	
C28	0.0991 (3)	0.3476 (5)	0.6623 (4)	0.0633 (15)	
H28	0.1032	0.3727	0.7148	0.076*	
C29	0.1030 (2)	0.4288 (5)	0.6018 (3)	0.0578 (14)	
C30	0.1133 (2)	0.5557 (5)	0.6152 (3)	0.0539 (13)	
C31	−0.0498 (3)	0.8056 (9)	0.5893 (4)	0.123 (3)	
H31A	−0.0428	0.7302	0.5647	0.148*	
H31B	−0.0546	0.8653	0.5490	0.148*	
H31C	−0.0898	0.8028	0.6203	0.148*	
C32	0.0092 (3)	0.8346 (8)	0.6421 (4)	0.0823 (19)	
C33	0.4142 (3)	0.7951 (8)	0.7368 (4)	0.098 (2)	
H33A	0.4389	0.7336	0.7111	0.117*	
H33B	0.4410	0.8656	0.7394	0.117*	
H33C	0.3735	0.8108	0.7071	0.117*	
C34	0.3974 (3)	0.7571 (5)	0.8182 (3)	0.0577 (13)	
C35	0.2721 (3)	0.3096 (6)	0.6249 (3)	0.0702 (16)	
H35	0.2602	0.2358	0.6447	0.084*	
C36	0.2733 (3)	0.3267 (7)	0.5437 (4)	0.0825 (19)	
H36	0.2634	0.2647	0.5091	0.099*	
C37	0.2888 (3)	0.4340 (8)	0.5155 (4)	0.0827 (19)	
H37	0.2901	0.4461	0.4608	0.099*	
C38	0.3029 (3)	0.5266 (6)	0.5663 (3)	0.0768 (17)	
H38	0.3121	0.6013	0.5462	0.092*	
C39	0.3033 (3)	0.5082 (5)	0.6456 (3)	0.0607 (14)	
H39	0.3140	0.5705	0.6796	0.073*	
C40	0.2881 (2)	0.3992 (5)	0.6772 (3)	0.0494 (12)	
C41	0.2861 (3)	0.3773 (5)	0.7645 (3)	0.0528 (12)	

### Atomic displacement parameters (Å^2^)

**Table:**

	*U*^11^	*U*^22^	*U*^33^	*U*^12^	*U*^13^	*U*^23^
N1	0.039 (2)	0.042 (2)	0.047 (2)	−0.0067 (17)	0.0019 (17)	0.0073 (19)
O1A	0.0632 (19)	0.0374 (17)	0.0361 (16)	−0.0078 (16)	−0.0019 (15)	0.0036 (15)
O1B	0.080 (2)	0.070 (2)	0.0413 (19)	−0.020 (2)	0.0041 (17)	0.0045 (19)
O2A	0.0510 (17)	0.0457 (19)	0.0379 (17)	−0.0074 (15)	−0.0017 (13)	0.0004 (15)
O2B	0.142 (4)	0.078 (3)	0.042 (2)	−0.011 (3)	−0.015 (2)	0.004 (2)
O3A	0.0490 (18)	0.080 (3)	0.0489 (19)	0.0077 (18)	−0.0111 (15)	0.006 (2)
O3B	0.168 (6)	0.104 (4)	0.111 (4)	0.003 (4)	−0.041 (4)	0.053 (4)
O7	0.0489 (19)	0.074 (2)	0.049 (2)	0.0051 (18)	0.0111 (15)	0.0233 (18)
O11A	0.0421 (16)	0.0542 (19)	0.0433 (18)	−0.0109 (15)	0.0012 (14)	0.0066 (15)
O11B	0.051 (2)	0.104 (3)	0.083 (3)	−0.031 (2)	−0.009 (2)	0.005 (3)
O13A	0.0613 (19)	0.0440 (19)	0.0367 (17)	−0.0014 (16)	0.0077 (14)	−0.0043 (15)
O13B	0.156 (4)	0.042 (2)	0.064 (2)	−0.020 (3)	0.016 (2)	−0.001 (2)
O19	0.0430 (18)	0.092 (3)	0.080 (2)	−0.0066 (19)	0.0115 (18)	0.017 (2)
C1	0.043 (2)	0.038 (2)	0.032 (2)	−0.001 (2)	0.0035 (19)	0.000 (2)
C2	0.047 (2)	0.041 (3)	0.042 (2)	−0.004 (2)	0.000 (2)	0.006 (2)
C3	0.045 (2)	0.046 (3)	0.048 (3)	0.008 (2)	−0.003 (2)	0.007 (2)
C4	0.042 (2)	0.048 (3)	0.051 (3)	0.006 (2)	0.002 (2)	0.001 (2)
C5	0.044 (2)	0.041 (2)	0.035 (2)	−0.003 (2)	0.002 (2)	0.005 (2)
C6	0.034 (2)	0.066 (3)	0.045 (3)	0.002 (2)	0.007 (2)	0.006 (3)
C7	0.052 (3)	0.047 (3)	0.033 (2)	0.006 (2)	0.003 (2)	0.008 (2)
C8	0.042 (2)	0.042 (2)	0.031 (2)	0.004 (2)	0.0007 (18)	0.001 (2)
C9	0.041 (2)	0.037 (2)	0.034 (2)	−0.002 (2)	0.0027 (19)	−0.005 (2)
C10	0.039 (2)	0.035 (2)	0.035 (2)	−0.0017 (18)	0.0003 (19)	−0.0051 (19)
C11	0.047 (2)	0.045 (3)	0.031 (2)	−0.008 (2)	−0.001 (2)	0.001 (2)
C12	0.041 (2)	0.056 (3)	0.042 (3)	0.000 (2)	0.004 (2)	0.001 (2)
C13	0.053 (3)	0.043 (3)	0.039 (3)	0.004 (2)	0.002 (2)	0.002 (2)
C14	0.044 (2)	0.038 (2)	0.036 (2)	−0.003 (2)	0.0031 (19)	0.004 (2)
C15	0.050 (3)	0.059 (3)	0.036 (2)	−0.001 (3)	−0.002 (2)	0.000 (3)
C16	0.052 (3)	0.055 (3)	0.039 (2)	0.002 (2)	−0.002 (2)	0.006 (2)
C17	0.057 (3)	0.138 (6)	0.063 (4)	0.006 (4)	−0.008 (3)	0.020 (4)
C18	0.053 (3)	0.079 (4)	0.056 (3)	0.017 (3)	0.002 (2)	−0.002 (3)
C19	0.042 (3)	0.067 (3)	0.054 (3)	0.000 (3)	0.002 (2)	0.004 (3)
C20	0.049 (3)	0.038 (3)	0.034 (2)	−0.005 (2)	−0.002 (2)	0.006 (2)
C21	0.061 (3)	0.056 (3)	0.069 (3)	−0.022 (3)	−0.007 (3)	0.019 (3)
C22	0.156 (7)	0.053 (4)	0.066 (4)	−0.032 (4)	0.017 (4)	0.003 (3)
C23	0.077 (3)	0.048 (3)	0.036 (3)	−0.018 (3)	−0.001 (2)	0.010 (3)
C24	0.089 (4)	0.085 (5)	0.065 (4)	−0.008 (4)	−0.007 (3)	−0.018 (3)
C25	0.129 (6)	0.095 (6)	0.091 (5)	−0.015 (5)	−0.009 (4)	−0.045 (5)
C26	0.096 (5)	0.067 (5)	0.128 (7)	−0.007 (4)	0.012 (5)	−0.040 (5)
C27	0.071 (4)	0.062 (4)	0.103 (5)	−0.005 (3)	0.012 (3)	−0.018 (4)
C28	0.061 (3)	0.062 (4)	0.067 (4)	−0.005 (3)	0.002 (3)	−0.009 (3)
C29	0.048 (3)	0.066 (4)	0.059 (3)	−0.005 (3)	−0.003 (2)	−0.011 (3)
C30	0.054 (3)	0.063 (4)	0.044 (3)	−0.006 (3)	−0.006 (2)	−0.002 (3)
C31	0.077 (4)	0.222 (10)	0.070 (4)	0.043 (6)	−0.015 (3)	0.011 (6)
C32	0.085 (4)	0.100 (5)	0.061 (4)	0.027 (4)	−0.014 (3)	0.013 (4)
C33	0.066 (4)	0.144 (7)	0.085 (4)	−0.027 (4)	0.021 (3)	0.029 (5)
C34	0.051 (3)	0.058 (3)	0.064 (3)	−0.021 (3)	0.009 (3)	0.005 (3)
C35	0.073 (4)	0.067 (4)	0.071 (4)	−0.008 (3)	0.012 (3)	−0.020 (3)
C36	0.083 (4)	0.100 (6)	0.064 (4)	−0.010 (4)	−0.003 (3)	−0.033 (4)
C37	0.083 (4)	0.116 (6)	0.049 (3)	0.009 (4)	−0.007 (3)	−0.010 (4)
C38	0.091 (4)	0.085 (5)	0.055 (4)	0.002 (4)	0.009 (3)	0.016 (4)
C39	0.070 (3)	0.064 (4)	0.048 (3)	0.007 (3)	0.010 (3)	−0.004 (3)
C40	0.054 (3)	0.052 (3)	0.042 (3)	−0.001 (2)	0.009 (2)	−0.009 (2)
C41	0.063 (3)	0.043 (3)	0.053 (3)	−0.002 (3)	0.014 (2)	−0.007 (3)

### Geometric parameters (Å, °)

**Table:**

N1—C6	1.456 (6)	C14—C20	1.551 (6)
N1—C20	1.459 (5)	C14—H14	0.9800
N1—C21	1.464 (6)	C15—C16	1.506 (6)
O1A—C23	1.352 (5)	C15—H15A	0.9700
O1A—C1	1.464 (5)	C15—H15B	0.9700
O1B—C23	1.206 (6)	C16—C17	1.304 (7)
O2A—C30	1.369 (5)	C17—H17A	0.9300
O2A—C2	1.445 (6)	C17—H17B	0.9300
O2B—C30	1.203 (6)	C18—H18A	0.9600
O3A—C32	1.354 (8)	C18—H18B	0.9600
O3A—C3	1.447 (5)	C18—H18C	0.9600
O3B—C32	1.165 (9)	C19—H19	0.9300
O7—C7	1.420 (5)	C20—H20	0.9800
O7—H7A	0.8200	C21—H21A	0.9600
O11A—C34	1.358 (5)	C21—H21B	0.9600
O11A—C11	1.445 (5)	C21—H21C	0.9600
O11B—C34	1.190 (6)	C22—C23	1.480 (8)
O13A—C41	1.342 (6)	C22—H22A	0.9600
O13A—C13	1.457 (5)	C22—H22B	0.9600
O13B—C41	1.197 (6)	C22—H22C	0.9600
O19—C19	1.216 (5)	C24—C29	1.385 (8)
C1—C10	1.519 (6)	C24—C25	1.389 (11)
C1—C2	1.520 (6)	C24—H24	0.9300
C1—H1	0.9800	C25—C26	1.360 (11)
C2—C3	1.506 (6)	C25—H25	0.9300
C2—H2	0.9800	C26—C27	1.356 (9)
C3—C4	1.525 (6)	C26—H26	0.9300
C3—H3	0.9800	C27—C28	1.394 (8)
C4—C19	1.538 (8)	C27—H27	0.9300
C4—C18	1.545 (7)	C28—C29	1.374 (8)
C4—C5	1.552 (6)	C28—H28	0.9300
C5—C6	1.528 (7)	C29—C30	1.467 (8)
C5—C10	1.570 (6)	C31—C32	1.494 (9)
C5—H5	0.9800	C31—H31A	0.9600
C6—C7	1.524 (6)	C31—H31B	0.9600
C6—H6	0.9800	C31—H31C	0.9600
C7—C8	1.522 (6)	C33—C34	1.479 (7)
C7—H7	0.9800	C33—H33A	0.9600
C8—C15	1.529 (6)	C33—H33B	0.9600
C8—C14	1.552 (6)	C33—H33C	0.9600
C8—C9	1.553 (6)	C35—C40	1.376 (7)
C9—C11	1.557 (6)	C35—C36	1.381 (8)
C9—C10	1.564 (6)	C35—H35	0.9300
C9—H9	0.9800	C36—C37	1.341 (10)
C10—C20	1.576 (6)	C36—H36	0.9300
C11—C12	1.538 (7)	C37—C38	1.377 (9)
C11—H11	0.9800	C37—H37	0.9300
C12—C16	1.501 (6)	C38—C39	1.350 (7)
C12—C13	1.507 (6)	C38—H38	0.9300
C12—H12	0.9800	C39—C40	1.378 (7)
C13—C14	1.544 (6)	C39—H39	0.9300
C13—H13	0.9800	C40—C41	1.490 (7)
			
C6—N1—C20	103.7 (3)	H15A—C15—H15B	108.1
C6—N1—C21	120.4 (4)	C17—C16—C12	124.5 (5)
C20—N1—C21	118.7 (4)	C17—C16—C15	124.2 (4)
C23—O1A—C1	115.6 (4)	C12—C16—C15	111.1 (4)
C30—O2A—C2	117.4 (4)	C16—C17—H17A	120.0
C32—O3A—C3	116.8 (5)	C16—C17—H17B	120.0
C7—O7—H7A	109.5	H17A—C17—H17B	120.0
C34—O11A—C11	114.4 (3)	C4—C18—H18A	109.5
C41—O13A—C13	117.6 (4)	C4—C18—H18B	109.5
O1A—C1—C10	106.4 (3)	H18A—C18—H18B	109.5
O1A—C1—C2	105.1 (3)	C4—C18—H18C	109.5
C10—C1—C2	115.4 (4)	H18A—C18—H18C	109.5
O1A—C1—H1	109.9	H18B—C18—H18C	109.5
C10—C1—H1	109.9	O19—C19—C4	122.2 (5)
C2—C1—H1	109.9	O19—C19—H19	118.9
O2A—C2—C3	112.5 (4)	C4—C19—H19	118.9
O2A—C2—C1	107.2 (3)	N1—C20—C14	109.1 (3)
C3—C2—C1	110.8 (4)	N1—C20—C10	103.3 (4)
O2A—C2—H2	108.7	C14—C20—C10	104.6 (3)
C3—C2—H2	108.7	N1—C20—H20	113.0
C1—C2—H2	108.7	C14—C20—H20	113.0
O3A—C3—C2	109.2 (3)	C10—C20—H20	113.0
O3A—C3—C4	107.5 (3)	N1—C21—H21A	109.5
C2—C3—C4	116.5 (4)	N1—C21—H21B	109.5
O3A—C3—H3	107.8	H21A—C21—H21B	109.5
C2—C3—H3	107.8	N1—C21—H21C	109.5
C4—C3—H3	107.8	H21A—C21—H21C	109.5
C3—C4—C19	109.8 (4)	H21B—C21—H21C	109.5
C3—C4—C18	108.6 (4)	C23—C22—H22A	109.5
C19—C4—C18	107.2 (4)	C23—C22—H22B	109.5
C3—C4—C5	110.8 (4)	H22A—C22—H22B	109.5
C19—C4—C5	111.7 (4)	C23—C22—H22C	109.5
C18—C4—C5	108.6 (4)	H22A—C22—H22C	109.5
C6—C5—C4	108.5 (4)	H22B—C22—H22C	109.5
C6—C5—C10	99.2 (3)	O1B—C23—O1A	122.5 (4)
C4—C5—C10	116.7 (4)	O1B—C23—C22	125.1 (5)
C6—C5—H5	110.6	O1A—C23—C22	112.4 (5)
C4—C5—H5	110.6	C29—C24—C25	119.3 (7)
C10—C5—H5	110.6	C29—C24—H24	120.3
N1—C6—C7	113.4 (4)	C25—C24—H24	120.3
N1—C6—C5	97.1 (3)	C26—C25—C24	120.8 (7)
C7—C6—C5	111.9 (4)	C26—C25—H25	119.6
N1—C6—H6	111.2	C24—C25—H25	119.6
C7—C6—H6	111.2	C27—C26—C25	120.5 (7)
C5—C6—H6	111.2	C27—C26—H26	119.7
O7—C7—C8	107.4 (4)	C25—C26—H26	119.7
O7—C7—C6	112.0 (4)	C26—C27—C28	119.5 (7)
C8—C7—C6	110.6 (3)	C26—C27—H27	120.2
O7—C7—H7	108.9	C28—C27—H27	120.2
C8—C7—H7	108.9	C29—C28—C27	120.7 (6)
C6—C7—H7	108.9	C29—C28—H28	119.6
C7—C8—C15	110.7 (3)	C27—C28—H28	119.6
C7—C8—C14	109.6 (3)	C28—C29—C24	119.1 (5)
C15—C8—C14	111.7 (4)	C28—C29—C30	123.3 (5)
C7—C8—C9	110.5 (3)	C24—C29—C30	117.6 (6)
C15—C8—C9	115.1 (4)	O2B—C30—O2A	122.2 (5)
C14—C8—C9	98.8 (3)	O2B—C30—C29	125.8 (5)
C8—C9—C11	105.6 (3)	O2A—C30—C29	112.0 (5)
C8—C9—C10	100.8 (3)	C32—C31—H31A	109.5
C11—C9—C10	123.0 (3)	C32—C31—H31B	109.5
C8—C9—H9	108.8	H31A—C31—H31B	109.5
C11—C9—H9	108.8	C32—C31—H31C	109.5
C10—C9—H9	108.8	H31A—C31—H31C	109.5
C1—C10—C9	115.8 (3)	H31B—C31—H31C	109.5
C1—C10—C5	113.2 (3)	O3B—C32—O3A	122.7 (6)
C9—C10—C5	105.3 (3)	O3B—C32—C31	127.8 (7)
C1—C10—C20	114.9 (4)	O3A—C32—C31	109.4 (7)
C9—C10—C20	104.4 (3)	C34—C33—H33A	109.5
C5—C10—C20	101.8 (3)	C34—C33—H33B	109.5
O11A—C11—C12	111.4 (4)	H33A—C33—H33B	109.5
O11A—C11—C9	115.0 (3)	C34—C33—H33C	109.5
C12—C11—C9	110.7 (4)	H33A—C33—H33C	109.5
O11A—C11—H11	106.4	H33B—C33—H33C	109.5
C12—C11—H11	106.4	O11B—C34—O11A	122.8 (4)
C9—C11—H11	106.4	O11B—C34—C33	124.4 (5)
C16—C12—C13	109.3 (4)	O11A—C34—C33	112.8 (5)
C16—C12—C11	105.6 (4)	C40—C35—C36	121.5 (6)
C13—C12—C11	110.9 (3)	C40—C35—H35	119.3
C16—C12—H12	110.3	C36—C35—H35	119.3
C13—C12—H12	110.3	C37—C36—C35	119.0 (6)
C11—C12—H12	110.3	C37—C36—H36	120.5
O13A—C13—C12	106.7 (4)	C35—C36—H36	120.5
O13A—C13—C14	111.9 (3)	C36—C37—C38	120.9 (6)
C12—C13—C14	110.1 (4)	C36—C37—H37	119.6
O13A—C13—H13	109.3	C38—C37—H37	119.6
C12—C13—H13	109.3	C39—C38—C37	119.6 (7)
C14—C13—H13	109.3	C39—C38—H38	120.2
C13—C14—C20	115.1 (3)	C37—C38—H38	120.2
C13—C14—C8	109.5 (3)	C38—C39—C40	121.5 (6)
C20—C14—C8	100.1 (3)	C38—C39—H39	119.3
C13—C14—H14	110.6	C40—C39—H39	119.3
C20—C14—H14	110.6	C35—C40—C39	117.5 (5)
C8—C14—H14	110.6	C35—C40—C41	119.9 (5)
C16—C15—C8	110.4 (3)	C39—C40—C41	122.6 (5)
C16—C15—H15A	109.6	O13B—C41—O13A	123.0 (4)
C8—C15—H15A	109.6	O13B—C41—C40	125.5 (5)
C16—C15—H15B	109.6	O13A—C41—C40	111.4 (4)
C8—C15—H15B	109.6		

### Hydrogen-bond geometry (Å, °)

**Table:**

*D*—H···*A*	*D*—H	H···*A*	*D*···*A*	*D*—H···*A*
O7—H7A···O19^i^	0.82	1.94	2.743 (5)	167

Symmetry codes: (i) −*x*, *y*, −*z*+2.
